# Environmentally sustainable smart cities and their converging AI, IoT, and big data technologies and solutions: an integrated approach to an extensive literature review

**DOI:** 10.1186/s42162-023-00259-2

**Published:** 2023-04-05

**Authors:** Simon Elias Bibri, Alahi Alexandre, Ayyoob Sharifi, John Krogstie

**Affiliations:** 1 School of Architecture, Civil and Environmental Engineering, Civil Engineering Institute, Visual Intelligence for Transportation , Swiss Federal Institute of Technology in Lausanne (EPFL), GC C1 383 (Bâtiment GC), Station 18, 1015 Lausanne, Switzerland; 2 Graduate School of Humanities and Social Science, Graduate School of Advanced Science and Engineering, Network for Education and Research on Peace and Sustainability (NERPS), Hiroshima University, 1-3-1 Kagamiyama, Higashi-Hiroshima, 739-8530 Japan; 3 Department of Computer Science, Norwegian University of Science and Technology, Sem Saelands Veie 9, 7491 Trondheim, Norway

**Keywords:** Environmentally sustainable smart cities, Smart cities, Sustainable cities, AI, AIoT, IoT, Big data, Environmental sustainability, Climate change

## Abstract

There have recently been intensive efforts aimed at addressing the challenges of environmental degradation and climate change through the applied innovative solutions of AI, IoT, and Big Data. Given the synergistic potential of these advanced technologies, their convergence is being embraced and leveraged by smart cities in an attempt to make progress toward reaching the environmental targets of sustainable development goals under what has been termed “environmentally sustainable smart cities.” This new paradigm of urbanism represents a significant research gap in and of itself. To fill this gap, this study explores the key research trends and driving factors of environmentally sustainable smart cities and maps their thematic evolution. Further, it examines the fragmentation, amalgamation, and transition of their underlying models of urbanism as well as their converging AI, IoT, and Big Data technologies and solutions. It employs and combines bibliometric analysis and evidence synthesis methods. A total of 2,574 documents were collected from the Web of Science database and compartmentalized into three sub-periods: 1991–2015, 2016–2019, and 2020–2021. The results show that environmentally sustainable smart cities are a rapidly growing trend that markedly escalated during the second and third periods—due to the acceleration of the digitalization and decarbonization agendas—thanks to COVID-19 and the rapid advancement of data-driven technologies. The analysis also reveals that, while the overall priority research topics have been dynamic over time—some AI models and techniques and environmental sustainability areas have received more attention than others. The evidence synthesized indicates that the increasing criticism of the fragmentation of smart cities and sustainable cities, the widespread diffusion of the SDGs agenda, and the dominance of advanced ICT have significantly impacted the materialization of environmentally sustainable smart cities, thereby influencing the landscape and dynamics of smart cities. It also suggests that the convergence of AI, IoT, and Big Data technologies provides new approaches to tackling the challenges of environmental sustainability. However, these technologies involve environmental costs and pose ethical risks and regulatory conundrums. The findings can inform scholars and practitioners of the emerging data-driven technology solutions of smart cities, as well as assist policymakers in designing and implementing responsive environmental policies.

## Introduction

Both of the leading global paradigms of urbanism—smart cities and sustainable cities—are facing unprecedented challenges pertaining to increasing urbanization, rapid urban growth, environmental degradation, and climate change. The escalating rate of urbanization is one of the special conundrums posed to all major cities worldwide, especially “mega-cities,” and has a strong connection to the most challenging and complex issues of our time. The 55% of the world's population living currently in urban areas is expected to rise to 70–75% by 2050 (UN 2022; UNDESA 2021). As engines of economic growth, cities are a major contributor to the global Greenhouse Gases (GHG) emissions. They consume more than 75% of the primary resources available globally, namely energy, fossil fuels, raw materials, food, and water. This rate of consumption is estimated to increase by 90 billion tons by 2050, compared to 40 billion tons in 2010 (Hajer et al. 2018). For example, urban areas consume 78% of world’s energy in industries based in cities (Hajer et al. 2018). Urban growth creates greater pressures on non-renewable energy sources and generate multiple conditions that pose significant environmental challenges to urban planners, policymakers, and decision-makers. Reducing reliance on fossil fuels contributes to reducing the climate change impacts, which are felt across the entire globe with all urban areas at risk.

Urbanization and urban growth exacerbate the wicked problems characterizing cities, whether those badging and regenerating themselves as sustainable/smart or those planning to become smart sustainable/sustainable smart, respectively. Consequently, they may jeopardize the endeavors being undertaken to achieve the long-term goals of environmental sustainability, as they put a high demand on the world’s natural resources and ecosystem services. With this situation being compounded by the increased uncertainty of the world, it is becoming more challenging for both smart cities and sustainable cities to reach the environmental targets of the Sustainable Development Goal (SDG) 11—without the adoption of the applied solutions of the Internet of Things (IoT), Big Data (Bibri and Krogstie 2020), and Artificial Intelligence (AI) technologies (Yigitcanlar et al. 2020). Indeed, smart cities and sustainable cities have recently started to integrate a wide variety of these data-driven technologies and green technologies and support those technological and environmental innovations capable of enabling and delivering environmentally sustainable urban development (Almalki et al. 2021; Gourisaria et al. 2023; Makani et al. 2022; Saravanan and Sakthinathan 2021).

In view of the above, the current circumstances require more effective responses and more holistic approaches. One of these, which has gained traction in more recent years, is the amalgamation of the aforementioned paradigms of urbanism to address and overcome the environmental challenges facing smart cities. The ultimate goal here is to accelerate the needed transition to environmental sustainability by harnessing the synergistic effects of the technologies and solutions of smart cities and sustainable development strategies in ways that improve the environmental performance of smart cities with respect to energy, transport systems, waste management systems, and building systems. These pertain to the emerging sustainable smart cities that tend to focus mainly on the environmental dimension of sustainability. One of the key factors that has led to the emergence of this paradigm of urbanism is the large body of research criticizing the weak connection between smart cities and sustainable cities and the fallacies and misunderstanding associated with smart cities in the context of environmental sustainability (Ahvenniemi et al. 2017; Angelidou et al. 2018; Bibri and Krogstie 2019; Evans et al. 2019; Stübinger and Schneider 2020; Toli and Murtagh 2020; Yigitcanlar et al. 2020). In a nutshell, it is often the case that smart city initiatives are driven by economic interests at the expense of environmental (and social) concerns (Trencher 2019). As a result, there has recently been an upsurge in research and practice outputs on environmentally sustainable smart cities (Al-Dabbagh 2022; Haarstad and Wathne 2019; Kim et al. 2021; Saravanan and Sakthinathan 2021; Shruti et al. 2019; Tripathi et al. 2022). This paradigm of urbanism is gaining momentum and hence evolving into a scholarly enterprise thanks to the integration of the core enabling technologies of smart cities, namely AI, IoT, Big Data, and Blockchain, as well as Wireless Sensor Network (WSN), Edge Computing, Digital Twins (DT), and cyber-physical systems.

In response to the climate emergency, coupled with the need to meet the aim of the decarbonization agenda (IPCC 2021; UNFCCC 2021) emerging sustainable smart cities are increasingly leveraging the collective expertise in AI, IoT, and Big Data—under AIoT—in developing innovative, immediate, and powerful solutions in the pursuit of environmental sustainability. Indeed, the more advanced AI models and techniques are needed to augment existing ICTs solutions with new capabilities in this regard. Broadly, AI describes computational capabilities inspired by human cognitive abilities (e.g., sensation, perception, language processing, learning, inference, reasoning, ad acting) to solve problems and achieve goals. As a subfield of computer science, AI is “the science and engineering of making intelligent machines” (McCarthy 2007) Still, there is no universal definition of AI or a definitive blueprint to build artificially intelligent machines (Cave et al. 2020; Clifton et al. 2020). Therefore, “it is necessary and beneficial to re-define the concept of AI and related terms to reflect the changing nature of AI development and applications in the era of Big Data” (Duan et al. 2019, p. 63). Concerning the relationship between AI, Big Data, and IoT, (Mohamed 2020) sheds light on the link between AI and IoT in relation to data management and analytics models, decision making, and human–machine interaction. (Bibri 2018a) discusses the link between IoT and Big Data in relation to sensor-based data collection, data processing platforms, and cloud and fog computing. Their convergence under AIoT entails that AI-based models and IoT devices collect colossal amounts of data from heterogenous sources, which are analyzed using AI-based data analytics techniques to predict patterns and make decisions. Overall, data-driven technologies are increasingly being integrated and applied in smart cities in the field of environmental sustainability (Bibri et al. 2023; Nishant et al. 2020; Sharifi et al. 2023a; Vinuesa et al. 2020; Yigitcanlar et al. 2020). However, AI is enshrined in public discourses as having positive connotations, while its environmental costs are often overlooked.

Against the preceding background, this study explores the key research trends and driving factors of environmentally sustainable smart cities and maps their thematic evolution. Further, it examines the fragmentation, amalgamation, and transition of their underlying models of urbanism as well as their converging AI, IoT, and Big Data technologies and solutions. Worth noting is that bibliometric analysis and evidence synthesis are both intended to bring together information from a wide range of sources and disciplines to inform public debates and decisions. These are indeed best served if policymakers have access to the relevant evidence base and a better understanding of the evolution of environmentally sustainable smart cities. In addition, research on the knowledge structure and trends of environmentally sustainable smart cities over different time periods remains very scant, adding to the scarcity of evidence of the convergence of AI, IoT, and Big Data technologies and solutions for environmental sustainability and climate change in the context of smart cities. In terms of contribution, the integration of bibliometric analysis and evidence synthesis is the main added value of this work, which resides in:

- Uncovering emerging technological trends in environmentally sustainable smart urbanism;
- Unpacking the evolutionary nuances of the field while shedding light on the rapidly evolving areas in it;
- Providing fertile insights into the development of the field to better serve scholars and practitioners with a vested interest in it by informing theory and practice;
- Identifying the knowledge structures of the field and the underlying environmental and technological components and their integration; and
- Understanding the underlying models of environmentally sustainable smart urbanism and its converging data-driven technologies and solutions.

This study is structured as follows: Sect. "Conceptual background" provides the background of the study. Sect. "Research review" introduces and defines the key relevant concepts. Sect. "Materials and methods" describes the materials and methods applied. Sect. "Results" presents the results, which are, in Sect. "Discussion", discussed and interpreted in respect of previous studies. Sect. "Recommendations for future research" provides recommendations for future research. This study ends, in Sect. "Conclusion", with conclusions.

## Conceptual background

### Smart cities

Numerous attempts have been undertaken to describe smart cities and their dimensions. They suggest a large number and wide variety of definitions and a plethora of directions to smart city development and transition (Albino et al. 2015; Toli and Murtagh 2020). This has resulted in “an alphabet soup of smart city approaches…, which has in turn generated a cacophony that has led to an exasperating confusion in the field of smart cities, despite the convergence on the idea that ICT constitutes a central focus in urban operational functioning and planning” (Bibri 2018b, p. 575). Nevertheless, recent work has moved past this conceptual quagmire by highlighting the emergence of a diverse landscape of smart cities (Karvonen et al. 2019). There are two working definitions for this study, which tend to complement one another. The first describes a smart city as “urban transformation that should aim to achieve a more environmentally sustainable city with a higher quality of life, [and] that offers opportunities for economic growth for all of its citizens… This transformation is currently enabled by various types of technologies…that are embedded into the city’s infrastructure system” (Toli and Murtagh 2020, p. 8). The second characterizes it as “a technologically and data–analytically advanced city that is able to monitor and understand its environment and citizens and to explore and analyze various forms of data to generate useful knowledge in the form of applied intelligence that can be used to solve different problems or to make changes to improve…the health of the city” (Bibri 2021a, p. 7). Indeed, data-driven urbanism, “the mode of production of smart cities,” (Kitchin 2016) has changed how the domains of transport, mobility, energy, environment, waste, lighting, and so on can be managed and planned (Kitchin et al. 2015; Nikitin et al. 2016). This has given rise to a new phenomenon known as data-driven smart cities (Dornhöfer et al. 2019; Kaluarachchi 2022; Kühne and Heidel 2021; Nelson and Neguriță, 2020; Sutherland and Cook 2017), where urban data are mostly collected via the connected IoT devices and analyzed using advanced AI models and techniques.

### Sustainable smart cities versus smart sustainable cities

Similarly, there are different approaches to, and definitions of, sustainable smart cities, depending on the technologies and strategies these integrated models for urban planning and development prioritize in response to the kind of problems and challenges they deal with in relation to environmental sustainability. Worth pointing out is that there is a fundamental difference between sustainable smart and smart sustainable cities. The former concept, which is the main focus of this study, refers to those smart cities (e.g., Barcelona, London, Singapore, Helsinki) that are striving to become environmentally sustainable based on integrating data-driven technologies and solutions with green technologies and strategies. The latter concept, which is mainly used in this study for comparative purposes, refers to those sustainable cities (e.g., Stockholm, Copenhagen, Amsterdam, Zurich) that are striving to improve and maintain their contribution to environmental sustainability on the basis of advancing their green technologies and strategies through data-driven technology solutions. Important to note is that these examples of cities are known for promoting certain aspects of environmental sustainability, and some of them are both smart and sustainable/sustainable smart. For example, Stockholm as a smart sustainable city aims to become climate positive by 2040 by using data-driven technologies to optimize or improve energy efficiency, air pollution, power grids, urban metabolism, and waste management (Bibri and Krogstie 2020). The difference between the two models of urbanism is also reflected in their early definitions. ITU (2015, 2016) defines a sustainable smart city as “an innovative city that uses ICT and other means to improve the quality of life, efficiency of urban operation and services, and competitiveness while ensuring that it meets the needs of present and future generations with respect to economic, social and environmental aspects.” A smart sustainable city is defined by (Höjer and Wangel 2015) Höjer and Wangel (2015, p. 10) “a city that meets the needs of its present inhabitants without compromising the ability for other people or future generations to meet their needs, and thus, does not exceed local or planetary environmental limitations, and where this is supported by ICT.”

## Research review

The focus of this study is on the environmental dimension of sustainability in smart cities. Several studies have, in more recent years, been conducted on emerging environmentally sustainable smart cities (Al-Dabbagh 2022; Kim et al. 2021; Makani et al. 2022; Saravanan and Sakthinathan  2021; Shruti et al. 2019; Singh et al. 2022; Tripathi et al. 2022). Indeed, they had tended to be limited until more recently compared to environmentally smart sustainable cities, which have gained popularity and become widely accepted since the mid-2010s. This is justified by the fact that sustainable cities (especially eco-cities) have, over the last four decades, been the central paradigm of sustainable urban development (Bibri 2021a, 2021b). As regards smart cities, (Stübinger and Schneider 2020) reveal in a data-driven systemic review that environmental sustainability will come to the fore in smart cities in the upcoming years, confirming the current trend due to the increasing importance of minimizing the required input of energy, waste, and water, as well as the heat and air pollution output. It follows that the review studies conducted on environmentally sustainable smart cities remain very scant compared to those performed on environmentally smart sustainable cities. Concerning the latter and their link with the convergence of AI, IoT, and Big Data technologies, there is only one review study that focuses on AI or AIoT applications for emerging smarter eco-cities as a model of environmentally smart sustainable urbanism (Bibri et al. 2023), and that employs and combines configurative, aggregative, and narrative syntheses. Further, most of the review studies conducted on the link between AI and smart cities address governance, planning, or sustainable development (Allam and Dhunny 2019; Navarathna and Malagi 2018; Nishant et al. 2020; Sharifi et al. 2023a). None of these deal with environmental sustainability from the perspective of AIoT in the context of smart cities. It follows that no bibliometric analysis has been carried out on emerging environmentally sustainable smart cities, nor review studies methodologically combining bibliometric analysis and evidence synthesis methods.

This comprehensive interdisciplinary review is the first of its kind with respect to combining smart cities, sustainable cities, environmental sustainability, and data-driven technologies as advanced forms of ICT. As such, it seeks to bring new insights into the flourishing field of environmentally sustainable smart cities and to extend knowledge on the underlying models of urbanism and technological areas by analyzing and synthesizing a plethora of studies of multiple disciplines. The research trend of environmentally sustainable urban development will undoubtedly continue in this direction. Hence, academic interest in the topic will increase as more attention is shifting toward the adoption of the data-driven technologies and solutions of smart cities to tackle the urgent challenges of environmental sustainability and the pressing threats of climate change. Therefore, it is of high relevance to explore the emerging interests, terminologies, developments, applications, dynamics, trends, and directions in the flourishing field of environmentally sustainable smart cities to help scholars, practitioners, and policymakers understand the underlying processes of its materialization, insertion, functioning, prevalence, appeal, and evolution.

## Materials and methods

This study employs a methodological framework as an innovative mix of two methods to capture both the comprehensiveness and contemporaneity of the topic on focus and the specificity and thoroughness of issues to be zoomed in. Accordingly, literature search, selection, and review procedures involve bibliometric analysis and evidence synthesis. The latter informs and expends on the former, which in turn frames and guides the latter, thereby producing a synergistic effect and an enhanced outcome—greater than the sum otherwise achieved by each of these methods if applied separately.

The bibliographic details of the scholarly literature on smart cities, sustainable cities, advanced ICT (IoT Big Data, and AI), and environmental sustainability and the associated keywords retrieved from the Web of Science (WoS) serve as the source of data for the bibliometric analysis. The rationale for selecting WoS among the available pool of academic research databases lies in its reputability in regard to its track record of indexing high-quality peer-reviewed studies related to the topic on focus and related comprehensive bibliographic data. These elements are crucial for conducting accurate analyses based on the bibliometric tools of VOSviewer. For each article, the WoS provides full record details, including cited references, making it possible to obtain co-citation analysis results of precision.

**Fig. 1:**
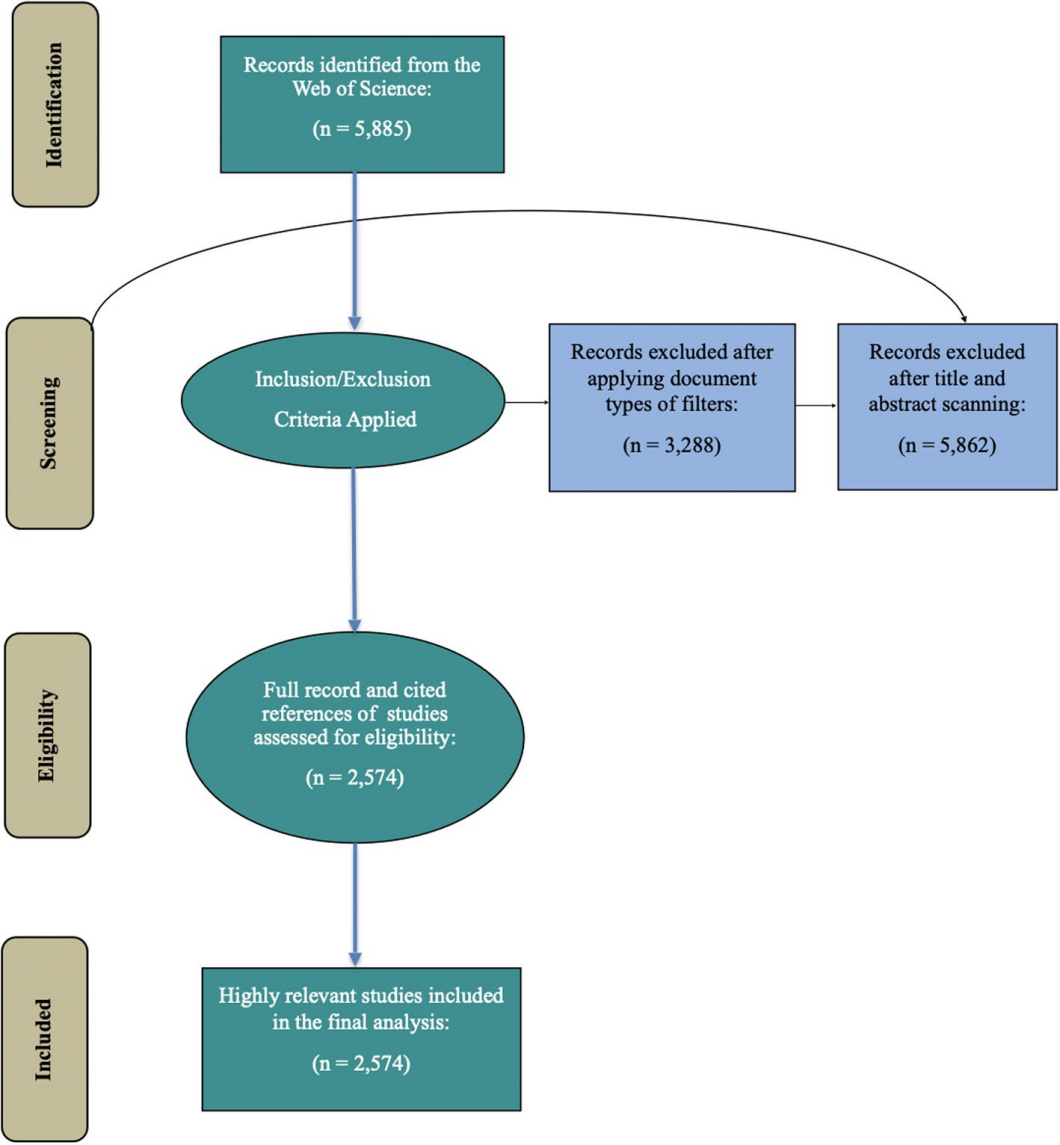
PRISMA flow diagram, four-phase flowchart of data extraction and filtration process

To retrieve relevant publications from the WoS, we developed a broad-based search string as a combination of different terms related to smart cities, sustainable cities, advanced ICT (IoT, Big Data, and AI), and environmental sustainability (see the Appendix). The search string returned 5885 documents, and the initial search was conducted in early January 2022 using different citation indexes of the WoS (i.e., AandHCI, ESCI, SCI-EXPANDED, SSCI). The articles, review articles, proceeding papers, book chapters, editorial materials, letters, and data papers published until the search date and written in English were considered for inclusion in the bibliometric analysis. Titles and abstracts of these documents were screened to select those focused on the relationships between smart city solutions/technologies and environmental sustainability. At the end, 2574 documents remained in the database. Bibliographic data of these documents, i.e., “Full Record and Cited References,” were downloaded from the WoS to be used for the bibliometric analysis. While the Preferred Reporting Items for Systematic Reviews and Meta-Analyses (PRISMA) (Fig. 1) is designed to help scholars employing these two review methods report the rationale for their review study, procedures, and final research outcome, its use has recently extended to include bibliometric studies (Moher et al. 2009; Page et al. 2021).

Various software tools have been developed for bibliometric analysis since the turn of the century ((Cobo et al. 2011). Some examples are VOSviewer, SciMAT, and CiteSpace. While having some unique features, each of these tools provides useful information on the structure of research fields and interactions between different components of academic papers (e.g., keywords, references, authors, journals, etc.). We used VOSviewer since its graphic outputs are more suitable for interpretation and its interface is user friendly. VOSviewer is an open-source software that can be downloaded from: https://www.vosviewer.com. The website also provides free access to user manuals and demo projects. We refer interested readers to the manuals for detailed step-by-step description of bibliometric analysis using VOSviewer.

The software was used to do different forms of analyses. These included term co-occurrence analysis (using the “fractional counting” counting method and setting “all keywords” as the unit of analysis), citation (setting “documents” as the unit of analysis), co-citation (using the “full counting” counting method, and setting “cited references,” “cited sources,” and “cited authors” as units of analysis), and bibliographic coupling (using the “full counting” counting method, and setting “organizations” and “countries” as units of analysis) (Van Eck and Waltman 2013). We used the term co-occurrence analysis to find out what is the thematic focus of existing research on environmentally sustainable smart cities. This analysis also allows understanding of how different topics are linked to each other. Based on interconnections between the terms, it is possible to identify thematic research clusters. To enhance the accuracy of the term co-occurrence analysis and avoid separate counting of anonymous terms, a thesaurus file was developed and added to the software database. It is possible to identify thematic research clusters based on interconnections between the terms. A thesaurus file was developed and added to the software database to enhance the accuracy of the term co-occurrence analysis and avoid separate counting of anonymous terms (e.g., IoT and Internet of Things). The graphic output of the term co-occurrence analysis and other analyses conducted in this study is a network of nodes and links, where node size is proportional to the number of times a given term has co-occurred with other terms in the network. Also, the link width is proportional to connection strength between two terms. Terms that have co-occurred frequently form thematic research clusters.

**Fig. 2:**
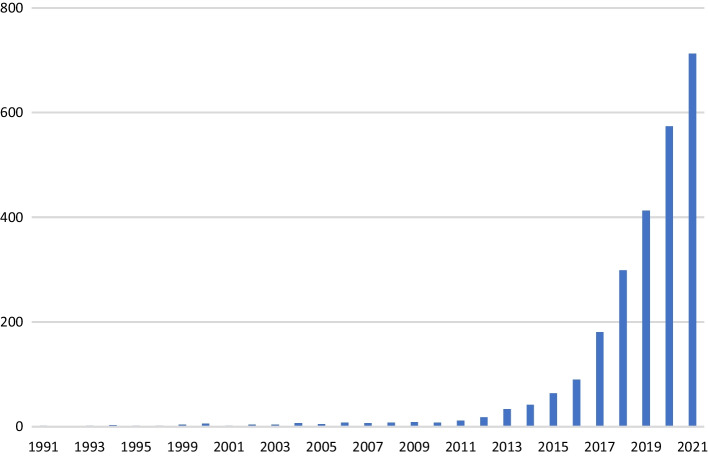
Number of publications on environmentally sustainable smart cities between 1991 and 2021

In this study we also aimed to explore the thematic evolution of the field over time. For that we divided the study period into three sub-periods, considering the number of publications in each period and also major milestones that may have influenced research on environmental sustainability in smart cities. The first milestone is 2015, when different international policy frameworks related to urban sustainability were introduced (e.g., Agenda 2030 and the New Urban Agenda). Other milestones before 2015 could have also been selected, but, as Fig. 2 shows, the number of publications till that date was relatively limited, not warranting a separate sub-period. The other milestone is 2020 when COVID-19 hits many cities worldwide. Accordingly, we examined these sub-periods: until 2015, 2016–2019, and 2020–2021. We did separate term co-occurrence analyses for each sub-period to examine how the field has evolved. Similar approach has been recently used to track the thematic evolution of other fields of research (Sharifi et al. 2023b; Sharifi and Khavarian-Garmsir 2020).

In addition, we combined bibliometric analysis with evidence synthesis. The latter brings together all relevant information on a research topic and high quality studies in a field. As Kirkevold (1997) notes, “far more information is extracted from a large literature by clearly describing the best evidence on a topic.” The evidence synthesis method entailed configurative synthesis and aggregative synthesis. These are complementary to one another and used in accordance with the identified themes. Thematic configuration involves interpretation during the process of synthesis to identify the big picture and construct the overall meaning—i.e., thematic synthesis. Aggregative synthesis entails interpretation after the process of synthesis to frame the findings—i.e., thematic summary. According to (Gough et al. 2017), aggregative synthesis adds up and leverages empirical evidence to make statements based on particular conceptual positions, thereby seeking evidence to inform decisions, while configurative synthesis interprets and arranges evidence and develops concepts, thereby seeking concepts for elucidation by means of new ways of understanding. Overall, this study intends to demonstrate the relevance and value of combining and integrating the two review categories for achieving and delivering better outcomes of knowledge.

## Results

The results collected after running the data in VOS viewer are analyzed as well as informed and expanded by bringing together all relevant information on the topic under study. Section "Publication trends and the predominant paradigms of urbanism" identifies the number of scholarly works devoted to the topic of emerging environmentally sustainable smart cities, the global shifts they interplay with, and their underlying models of urbanism. Section"Thematic focus areas and their transition toward environmentally sustainable smart cities" delves into the thematic focus areas related to smart cities, sustainable cities, environmental sustainability, and advanced ICT, as well as their transition toward environmentally sustainable smart cities and their converging IoT, Big Data, and AI technologies and solutions. It presents the findings for each of the three time periods that are used to categorize the publication years, as well as synthesizes multiple studies reporting on the topical subjects and issues related to such transition and convergence.

### Publication trends and the predominant paradigms of urbanism

#### Overview of research trends

Smart sustainable/sustainable smart cities are relatively new urban phenomena whose emergence has resulted from the convergence of a set of intertwined societal factors and technological trends, notably the rise of urbanization, the diffusion of sustainability, the dominance of ICT, and the shift in science and technology (big data science and analytics). These congeries of forces also shape and reshape their insertion, functioning, expansion, and continuation as currently predominant paradigms of urbanism. This has led to a plethora of streams of research, which have, since the mid-2010s, begun to rapidly evolve compared to the previous period starting from the early 2010s, as shown in Fig. 2. The focus of this study being on research and publications since 1991 is justified by the fact that the four major global shifts: urbanization, sustainability, ICT, science and technology, have emerged and become prevalent in different periods of history to converge under what is known as smart sustainable cities and shortly afterwards sustainable smart cities. From 1991, a few years after the publication of the Brundtland Report (UN 1987) and thus the adoption of sustainability in urban planning and development—until the late 1990s, research was scant in the area of smart cities, the first strand of urbanism that constitutes the notion of sustainable smart cities. During this period, ICT was still in the early stage of its development toward producing urban environments that later became quite different from what people in cities had experienced before then. The idea of smart cities was a science fiction, for much of the twentieth century, but quite suddenly the prospect of smart cities fast became the new reality thanks to the massive proliferation of relatively intelligent computable devices across multiple scales (Batty et al. 2012). (Cugurullo 2018.) examines the origins of the synergy between urban development and technological development characterizing the phenomenon of smart cities. These evolutionary processes of smart cities justify the start of the mapping of data in relation to the bibliometric analysis, as depicted in Fig. 2. Between 1991 and 2005, research on smart cities as an academic discourse was still in its infancy, thereby the status of academic interest in the topic compared to later periods.

The widespread popularization of the concept of smart cities due to the rapid rise of ICT since the early 2000s became associated with the rapid rise of urbanization since the early 2010s given the synergy between these two major global shifts. (Townsend 2013) portrays urban growth and ICT development as a form of symbiosis. Shortly afterwards, the link between advanced ICT and increasing urbanization became widely recognized in light of the report published by the United Nations on the SDGs in 2015 and its updated versions afterwards, especially SDG 11: Sustainable Cities and Communities. As a consequence, smart cities have evolved from ways of automating routine functions across urban systems (transport, traffic, energy, building, etc.) to ways of monitoring, analyzing, controlling, regulating, and governing the domains of urban life to improve efficiency, sustainability, and resilience. During the period 2011–2021, the increase in the quantity of the published works followed different patterns with respect to smart cities and their incorporation of the environmental objectives of sustainable development in their strategies (Al Nuaimi et al. 2015; Batty et al. 2012; Bibri 2018b, 2019; Correia and Wuenstel 2011; Nam and Pardo 2011; Neirotti et al. 2014; Nikitin et al. 2016). Similar patterns were followed concerning sustainable smart cities (Al-Dabbagh 2022; Kim et al. 2021; Perera et al. 2017; Saravanan and Sakthinathan 2021; Shruti et al. 2019), as well as smart sustainable cities (Bibri 2018a; D'Amico et al. 2020; Evans et al. 2019; Höjer and Wangel 2015; Kramers et al. 2014; Martin et al. 2018; Thornbush and Golubchikov 2019). As illustrated in Fig. 2, the increase in the quantity of the published works related to the convergence of smart cities, sustainable cities, environmental sustainability, and advanced ICT (especially data-driven technologies) followed different patterns during the period 2011–2021. The slight decrease in the number of publications during the period 2020–2021 could be attributed to the effects of COVID-19 on the scholarly research activities in the field and thus the published works. Moreover, 2016–2017 witnessed the highest number of publications on smart sustainable cities/sustainable smart cities due to several intertwined factors. These include the global response to the SDGs agenda and especially the SDG 11, the increasing criticisms of the fragmentation of smart cities and sustainable cities, and the rapid advancement of ICT (especially AI/ML, IoT, and Big Data).

#### The marked influence of sustainable cities on smart cities

As regards sustainable cities, the second strand of urbanism that constitutes the notion of sustainable smart cities, they became prevalent and evolved into the leading paradigm of sustainable urban development during the period 1991–2010 (Wheeler and Beatly 2014; Whitehead 2003; Williams 2009)—when the area of smart cities was still in infancy research and early development. While sustainable cities have been around for more than four decades or so (Bibri 2020; Jabareen 2006; Rapoport 2014), it is not until the early 2000s that they became the most preferred response to sustainability challenges and thus a powerful and more established academic and policy discourse (see Bibri and Krogstie 2017 for a detailed review). As a result, the high relevance and value of the findings generated from the research conducted in the field of sustainable cities have increased scholarly interest in the topic of smart sustainable cities as a strategic and holistic approach to sustainable urban development. This is manifested in not only the number of publications that skyrocket between 2015 and 2021, but also in the policy and public discourses reflected in the numerous documents and reports of international organizations, including:

- United Nations (UN)
- United Nations Human Settlement Programme (UN-HABITAT)
- United Nations Economic Commission for Europe (UNECE)
- European Commission (EC)
- Organization for Economic Co-operation and Development (OECD)
- United Nations Environment Program (UNEP)
- International Telecommunication Union (ITU)
- Environment Agency Austria (EAA)

In more recent years, for example, a global UN initiative called “the United for Smart Sustainable Cities (U4SSC),” which is coordinated by UN-habitat, UNECE, and ITU and supported by a number of UN agencies and other international organizations, aim to guide sustainable cities and communities “to become smarter and more sustainable while accelerating their digital transformation” toward achieving the SDGs (ITU 2022).

Urban politics and policy around the world are now infused with the language of sustainable smart/smart sustainable urbanism, and a complex tapestry of urban visions can be formed and a multitude of exemplary urban initiatives can be found. The subject of sustainable smart/smart sustainable cities has become very fascinating and enticing for scholars, practitioners, and policymakers, providing tremendous opportunities to address sustainable development challenges, spur groundbreaking research and innovation, and monitor progress in SDGs targets. As regards the latter, for example, (Marsal-Llacuna et al. 2015) note that urban monitoring started in the early 1990s after the development of the many indicators taken from sustainable cities to address smart city initiatives. (Al-Nasrawi et al. 2016) propose a multidimensional model that can evaluate the smartness level of a city and combines its sustainable and smart dimensions. The transformative processes enabled by sustainable cities have impacted the development of smart cities in a variety of ways. One of which is the incorporation of environmental indicators, targets, and goals in their development strategies and the integration of green technologies and sustainable design strategies with their digital and physical infrastructures. Smart urbanism—is about rebuilding cities through integrating digital technologies with spatial forms and networked infrastructures, which is “being represented as a unique emerging ‘solution’ to the majority of problems faced by cities today (Marvin et al. 2015). Barcelona as one of the leading smart cities in Europe defined a new development model for a healthy and hyper-connected city with zero emissions, a significant objective “where the environment, urban planning, and ICT infrastructures are fully integrated” (Mora et al. 2021, p.4).

Based on the extant literature on smart cities, which is consistent with Fig. 2, it is evident that the year 2011 marked the early phase of the convergence of smart cities and sustainable cities as a result of many smart cities in Europe starting to adopt the environmental strategies of sustainable cities. For example, Barcelona and London were the first European cities to heavily invest in ICT infrastructure and implement smart technologies in the early 2010s to improve their performance in relation to transport, energy, environment, and waste (Nikitin et al. 2016; Sinaeepourfard et al. 2016). Barcelona has made a strong commitment to globally becoming a show-case in environmentally sustainable urban (Bolici and Mora 2016), bringing IoT and Big Data to life. In a detailed case study analysis Bibri and Krogstie (2020) examine how smart urbanism is practiced and justified in Barcelona and Stockholm with regard to the development and implementation of innovative applied solutions for environmental sustainability. As Batty et al. (2012) pointed out a decade ago, smart cities can only badge themselves as such, “if there are intelligence functions that are able to integrate and synthesize data to some purpose, ways of improving the efficiency, equity, sustainability, and the quality of life in cities,” and research on smart cities will go beyond their instrumentation to include the way the latter opens up dramatically different forms of how cities will be operated and organized. All in all, the period 2011–2016 marked the materialization, expansion, and adoption of different applied technology solutions in the systems and domains of smart cities in the field of environmental sustainability, paving the way for the emergence of environmentally sustainable smart cities. This roughly started from 2017 onwards (Kim et al. 2021; Perera et al. 2017; Saravanan and Sakthinathan 2021; Shruti et al. 2019). This year marked an era where the integration of smart technologies and sustainable strategies became the mainstay of the future forms of urban development.

### Thematic focus areas and their transition toward environmentally sustainable smart cities

This subsection explores the thematic focus of research on sustainable smart cities/smart sustainable cities, and discusses how and why it has changed and evolved over time. It is broken down into three different subheadings, each of which focuses on a different time period, namely 1991–2015, 2016–2019, and 2020–2021. After providing an overview of the overarching topical themes, the subsequent subsections will focus on each of the distinct periods covering different strands of research.

#### Overall thematic focus and structure

**Fig. 3:**
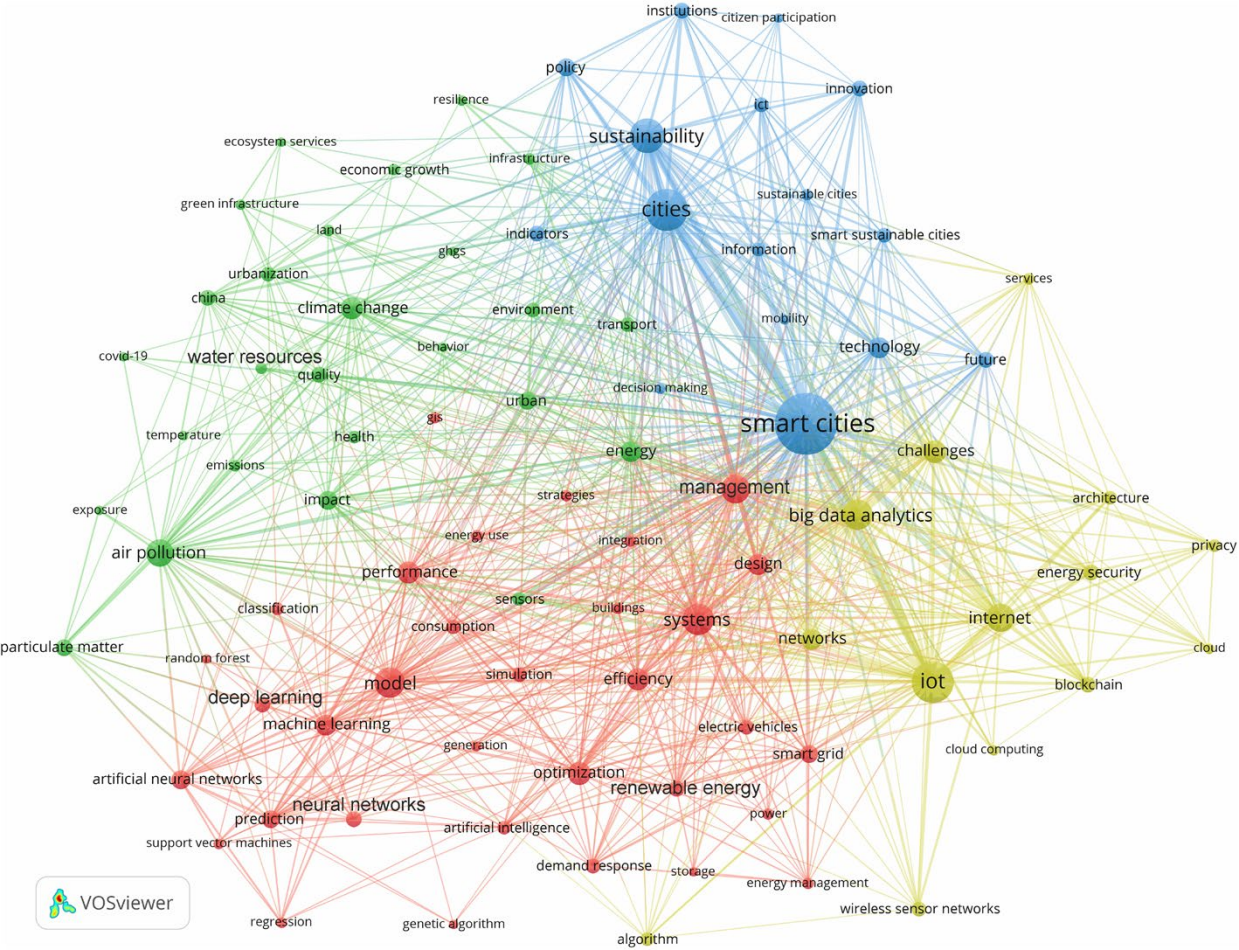
Overall thematic focus and structure from 1991–2021

As demonstrated in the preceding section, academic interest in the integration of smart cities and sustainable cities and their solutions and strategies has resulted in a significant increase in research and findings on a wide scope of topics (driven by facts and specifics) and themes (dealing with the big picture and overall meaning). The main ideas are outlined in the analysis performed on the different publications associated with the period 1991–2021. These publications are categorized into, as shown in Fig. 3, four distinct clusters: red, green, blue, and yellow, which are essentially closely linked due to the nature of sustainable smart cities being an integrated paradigm of urbanism. The Green cluster comprises publications focusing on issues related to environmental impacts, environmental sustainability, climate change, air pollution and quality, water resources, green infrastructure, ecosystem services, urbanization, land use, and so on. The Red cluster consists of publications targeting the implementation of the data-driven technologies and solutions of smart cities that could have positive impacts on environmental sustainability (e.g., through the optimization of renewable energy and smart grid systems). Several AI techniques are applied together with the IoT and Big Data technologies in smart cities in the field of environmental sustainability, including Artificial Neural Network (ANN), Support Vector Machine (SVM), Genetic Algorithm (GA), and Linear Regression (LR). The Yellow cluster represents publications covering the underlying technological infrastructures, architectures, platforms, systems, models, and networks of smart cities. Lastly, the Blue cluster is dedicated to publications focusing on broad issues related to smart cities and sustainable cities and their synergistic urban, technological, environmental, and institutional aspects. It also includes publications on smart sustainable cities as a more prevalent paradigm of urbanism than sustainable smart cities.

#### On the fragmentation and amalgamation of smart cities and sustainable cities: an environmental perspective

There is a long-standing criticism of the extreme fragmentation of the landscapes of smart cities and sustainable cities and its implications for hampering progress toward achieving the environmental targets of the SDG 11. Smart cities and sustainable cities are divergent on many aspects and hence not necessarily synonymous with each other (Ahvenniemi et al. 2017; Bibri and Krogstie 2017; Colding et al. 2020; Machado Junior et al.; Martin et al. 2018; Perng et al. 2018; Yigitcanlar et al. 2019). Therefore, recent research has focused on the shift of smart cities toward sustainable smart cities in the attempt to rebalance the three dimensions of sustainability (Haarstad and Wathne 2019; Machado Junior et al. 2018; Martin et al. 2018). This is due to the large body of research showing that existing smart cities remain disproportionately driven by economic interests and motives and thus lack the ability to address environmental (and social) concerns (Bina et al. 2020; Cugurullo 2018; Datta 2018; Kaika 2017; Perng et al. 2018; Shelton et al. 2015; Vanolo 2016; Verrest and Pfeffer 2019).

In response, smartness and sustainability have become highly linked as part of the recent research on new approaches to sustainable development. A comprehensive analysis of city labels shows that smart cities and sustainable cities are the most frequently co-occurring ones (Schraven et al. 2021). Under the scope of this study, data-driven smart urbanism is increasingly providing a plethora of applied innovative solutions to deal with the environmental pathologies pertaining to energy consumption, pollution, traffic congestion, mobility ineffectiveness, hazardous waste, unsustainable material, resource depletion, biodiversity loss, and climate change crisis. This is evidenced by a number of topical studies covering these topics (Almalki et al. 2021; Aymen and Mahmoudi 2019; Kim et al. 2021; Nishant et al. 2020; Perera et al. 2017; Saravanan and Sakthinathan 2021; Singh et al. 2022; Stübinger and Schneider 2020; Yigitcanlar et al. 2020). However, smart cities may fail to contribute to the agenda of environmental sustainability due to the incongruence of smart city policies and CO2 emissions. Furthermore, in a comprehensive state-of-the-art review of data-driven smart sustainable cities (Bibri 2021a) corroborates that sustainable urbanism will change “in fundamental and irreversible ways” due to the increasing adoption of the IoT and Big Data technologies, which are providing new capabilities for monitoring, managing, planning, and governing sustainable cities to advance and sustain their contribution to the goals of environmental sustainability. Especially, “eco-city projects are increasingly capitalizing on data-driven smart technologies to implement environmental…reforms. This is being accomplished by combining the strengths of smart cities and eco-cities and harnessing the synergies of their solutions and strategies in ways that enable eco-cities to improve their performance with respect to sustainability” (Bibri 2021a).

The growing interest in the innovative technologies and solutions of smart cities and their potential to tackle the environmental challenges of sustainability has resulted in an increase in research on a range of topics and themes related to smart sustainable cities in the context of environmental sustainability. This is illustrated in Fig. 3, which highlights the most prevalent models for urban planning and development and their environmental and technological dimensions that have evolved over time. As a result of these urban dynamics and their influence on the emergence of environmentally sustainable smart cities, the number of publications focusing on the underlying technologies, solutions, strategies, and policies have been on the rise since 2011, as illustrated in Fig. 2. The essence of this paradigm of urbanism lies in increasing and sustaining the contribution of smart cities to the goals of environmental sustainability thanks to the convergence of their IoT, Big Data, and AI technologies and solutions. This relates to large-scale urban computing and intelligence systems, which entail “connecting unobtrusive and ubiquitous sensing technologies, advanced data management and analytics models, and novel visualization methods to structure intelligent urban computing systems for smart cities” (Liu et al. 2017, p. 675). Based on a case study analysis of the innovative potential of urban computing and intelligence in the planning of smart sustainable/sustainable smart cities, Bibri (2021c) concludes that the “fast-flowing torrent of urban data, coupled with its analytical power, is of crucial importance to the effective planning and efficient design of this integrated model of urbanism. This is enabled by the…data-driven and model-driven decision support systems associated with urban intelligence…functions…that improve sustainability, efficiency, and resilience.”

#### Evolution of structures, thematic focus, urbanism models, and data-driven technologies

The timelines were used to map and categorize various events into three main periods of time. They were based on a set of several intertwined factors, including the inception of the notion of sustainable development and the emergence of sustainable cities, the rapid progress in technological development and the rise of advanced ICT, the prospect of smart cities fast becoming the new reality, the increasing rate of urbanization and its unintended consequences, the rising concerns of environmental sustainability and climate change, the deployment of ICT infrastructure on a citywide scale, the growing consensus on the pertinence of the integration of smart cities and sustainable cities, and the enticing features of the subject of environmentally sustainable smart cities as manifested in the variety and number of actors involved in the academic and practical aspects of the endeavor.

##### Period 1 (1991–2015):

**Fig. 4:**
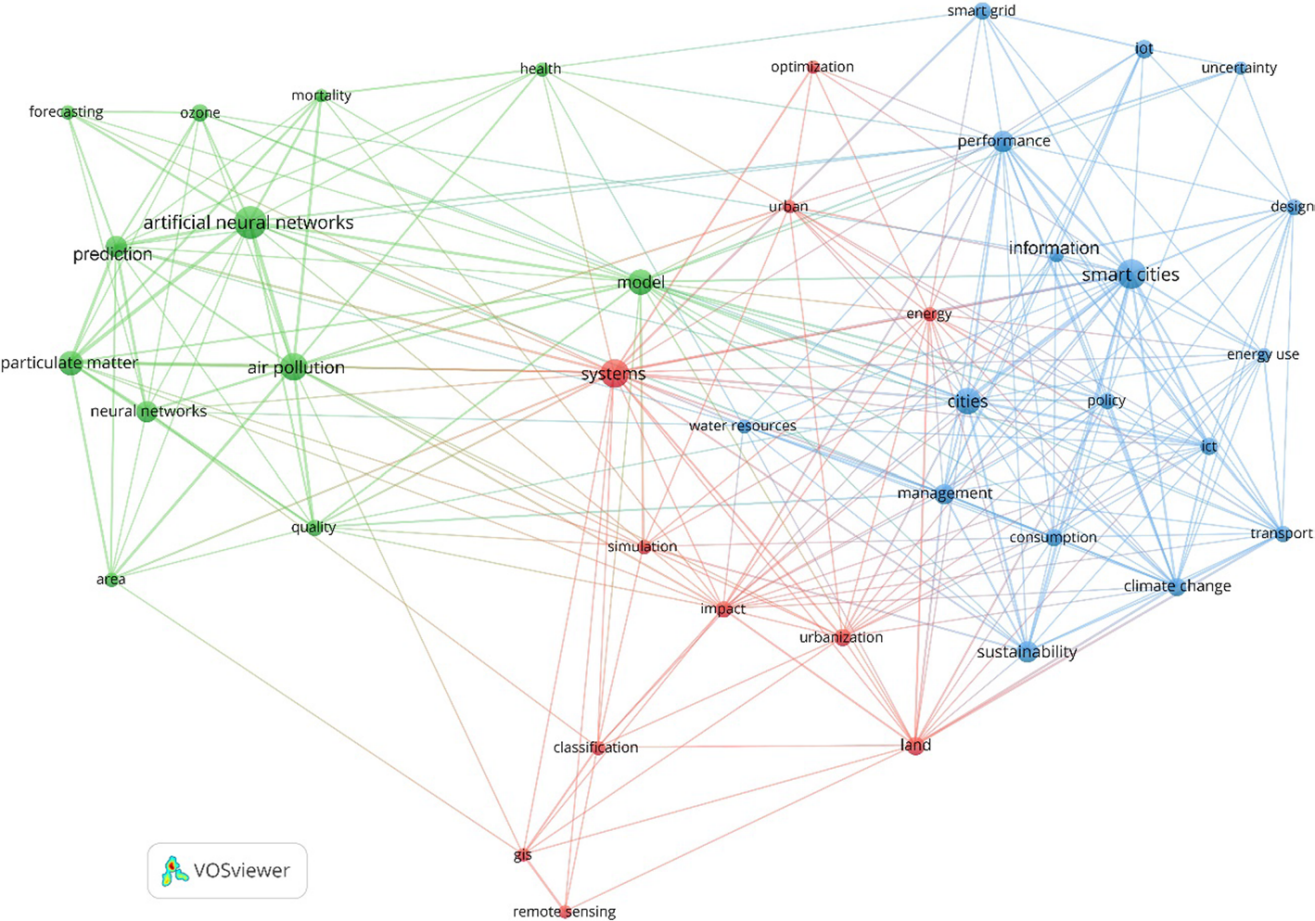
Evolution of environmentally sustainable smart cities during the First Period (1991–2015)

*Evolution of environmentally sustainable smart cities.* Figure 4 depicts the findings for the first period (1991–2015). While the concept of smart cities was introduced in 1994 (Dameri and Cocchia 2013), it is not until 2010 that the number of publications and scientific writings on the topic began to increase, following the emergence of smart city projects supported by the European Union (Jucevičius et al. 2014). In other words, while it can be traced back to the smart growth movement of the 1990s (Harrison and Donnelly 2011), it is not until recently that it came to be widely adopted in urban planning and development in response to this movement (Batty et al. 2012). This period marked the launch of many different specialized and interdisciplinary international journals on the topic of smart cities and their scientific, technological, and environmental research strands. This brought attention to the challenges of environmental sustainability and the potential of smart technologies and solutions among scholars, academics, and practitioners to overcome some of these challenges. The aim and scope of early journals revolved around the fundamental and applied research focused on theorizing, investigating, designing, developing, and implementing smart, resilient, and low-carbon cities. Accordingly, these journals promoted research and innovation in numerous areas, including smart cities and environments, ICT and intelligent infrastructures, smart grids, energy efficient and low/zero carbon buildings, climate change mitigation and adaptation, environmental risks and impacts, decision support systems for urban management, urban simulation models and optimization methods, and the IoT and Big Data applications in smart cities, and AI and ML applications for environmental sustainability and climate change, but to name a few. This is reflected across the three clusters as to the emphasis of the published academic work addressing the role of the emerging technologies and solutions of smart cities in optimizing energy efficiency across various domains and thereby mitigating pollution levels, among others.

**Fig. 5:**
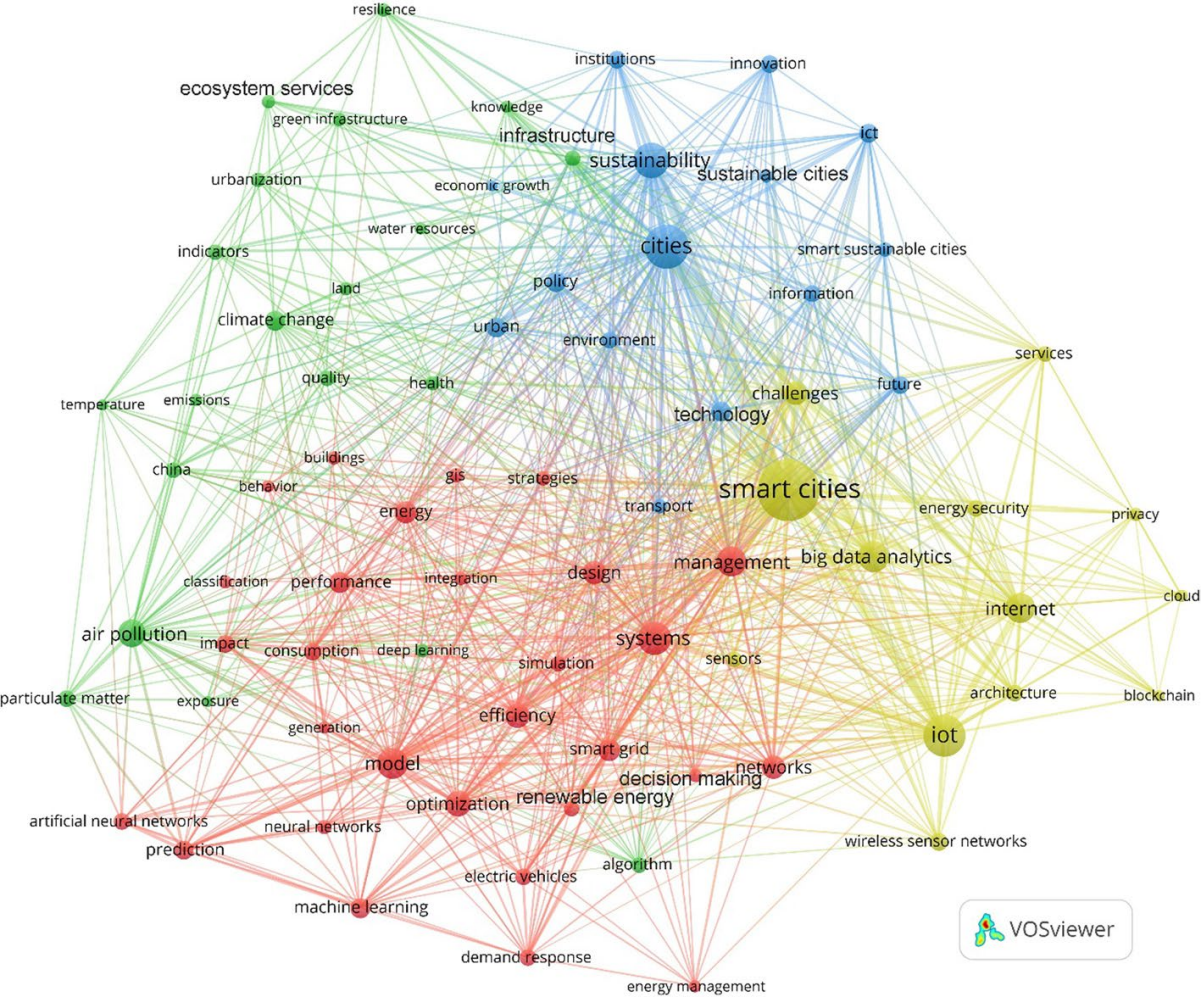
Evolution of environmentally sustainable smart cities during the Second Period (2016–2019)

**Fig. 6:**
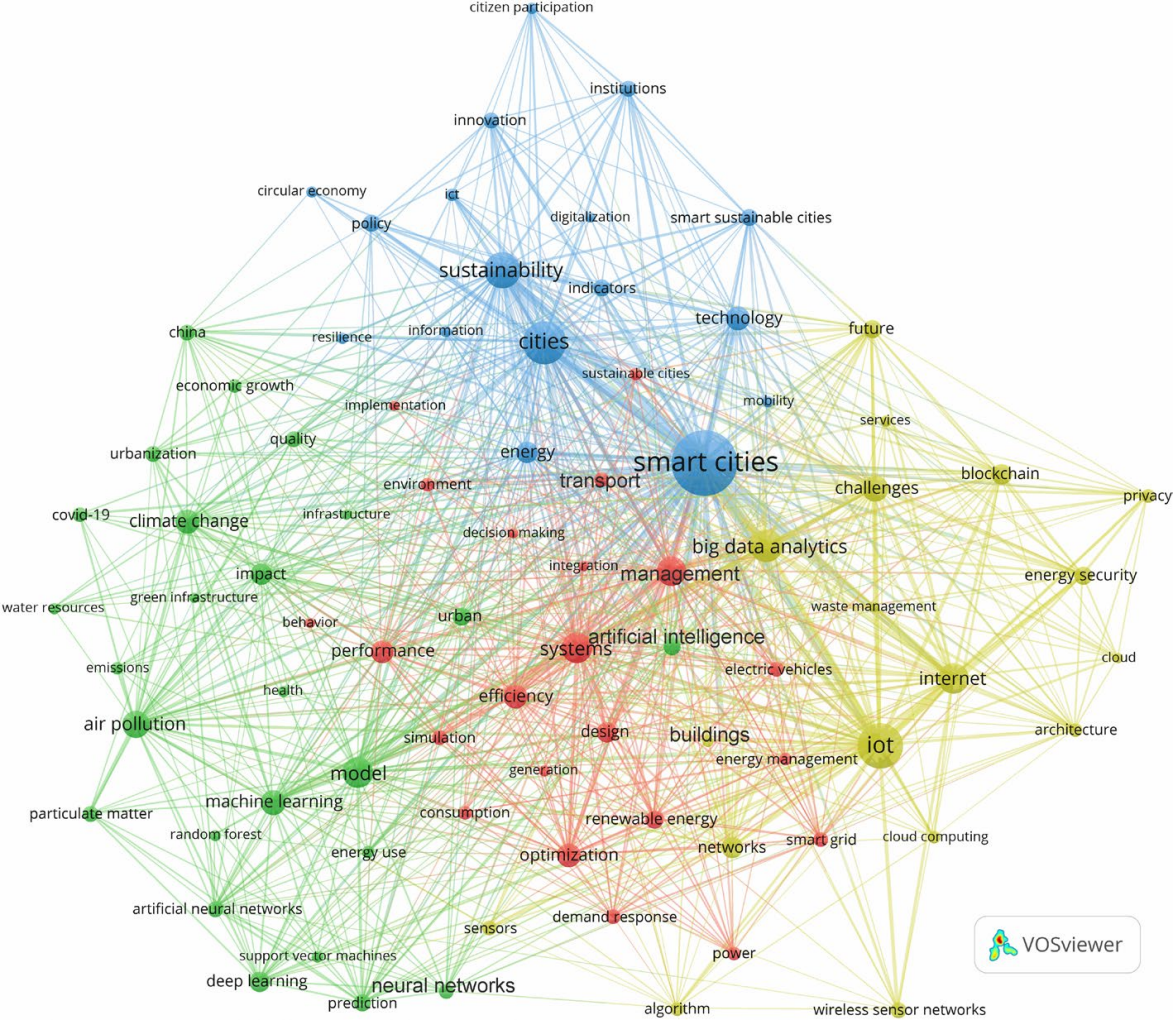
Evolution of environmentally sustainable smart cities during the Third Period (2020–2021)

The Green cluster in Fig. 4, which highlights environmental issues and impacts related to air pollution, air quality, particulate matter, ozone, and health during the first period, shows the early use of AI and a few of its models and techniques in the field of environmental sustainability in regard to forecasting and prediction. Climate change, energy consumption, transport, water resources, and other areas of environmental sustainability (the Blue cluster) were associated with the early smart city technologies and solutions as applied to optimization, management, and efficiency (i.e., urbanization and land use) (the Red cluster). This is clearly different from the findings indicated in Figs. 5 and 6 pertaining to the second and third periods, respectively. During the first period, smart grids were still an evolving topic in the context of smart cities. There were major barriers to the development of smart grids (see Brown and Zhou 2013 for a comprehensive review), policy issues related to sustainable smart grids (see Brown and Zhou 2012 for an early policy framework), and significant challenges for their implementation that needed to be addressed in the early 2010s. This included enhancing efficiency and renewables with smart grids and policies (Brown 2014). In particular, policy has a primary role in aligning and mobilizing different stakeholders in the same direction, and hence, it is more important than technology itself in the context of emerging smart sustainable/sustainable smart cities. Most of the smart technologies and solutions deployed during and after the first period were only plasters that failed to address the complex challenges of environmental sustainability. This means that environmental policies “must be put into place to maximize the benefits of these solutions through more effective measures…and ensure that progress is made in any area of environmental sustainability where innovative solutions need to be implemented” (Bibri and Krogstie 2020, p. 41).

Furthermore, the number of publications on emerging smart sustainable cities (not sustainable smart cities) indicates that while the interlinked developments of environmental sustainability, advanced ICT, and urbanization have converged under smart sustainable cities before the end of this period, it is not until the publication of the report on the SDGs (UNSDG 2015) that this convergence occurred regarding sustainable smart cities. The reasons for this occurrence will be explained as part of the analysis and synthesis of the first part of the second period. In this light, Fig. 4 mainly illustrates the incipient stage of the convergence of environmental sustainability, smart cities, and sustainable cities during the period 2010–2015. This clearly outlines scant thematic areas up until 2005 and limited thematic areas up until 2011 pursued by most publishers. The number of publications started to rise in a more or less systematic way during the period 2011–2015. Still, the amalgamation of smart cities and sustainable cities is not visible in Fig. 4 because environmentally sustainable smart cities started to emerge after this period (Kim et al. 2021; Perera et al. 2017; Saravanan and Sakthinathan 2021; Shruti et al. 2019). Toward the end of the period 1991–2015, research in the area of sustainable smart cities was still in its infancy but rapidly evolving.

##### Period 2 (2016–2019):

*Evolution of environmentally sustainable smart cities*. As highlighted in Fig. 5, the second period (2016–2019) marked the widespread popularization of the notion of smart sustainable/sustainable smart cities and subsequently the emergence of the early practical initiatives of both paradigms of urbanism in light of the merger of the landscapes of smart cities and sustainable cities. This indicates the wide recognition of the substantive effects of smart technologies on the future forms of urban planning and development. The areas concerned in this regard are, as shown in the Green cluster, climate change, land use, air pollution, air quality, particulate matter, GHG emissions, green infrastructure, resilience, water resources, and ecosystem services. Consequently, as shown in the Blue cluster, there was a marked increase in academic interest in the topics of sustainable cities, smart sustainable cities, and environmental sustainability, as well as the multiple connections between cities, the environment, and ICT from a general perspective. Such increase also involved smart cities and their relationship to these topics (the Yellow cluster). Still, the main focus of smart cities in the early phase of the second period was on the development and implementation of advanced ICT across urban systems and domains. The increased academic interest in those topics and their relationships reflects the influence of the SDGs agenda, the response to the criticism of the fragmentation of smart cities and sustainable cities, and the wide adoption of advanced technologies in various urban domains. In particular, international organizations and governmental institutions increased their support for the adoption of innovative solutions to address and overcome the challenges of environmental sustainability. Concurrently, as illustrated in the Yellow cluster, there was an unprecedented upsurge in research in the field of smart cities and their advanced technologies, namely IoT, Big Data Analytics, Cloud Computing, Blockchain, sensor, and WSN.

In parallel, a plethora of smart-city initiatives were developed and implemented across different geographies (Cowley et al. 2017; Datta 2018; Dowling et al. 2019; Fernandez-Anez et al. 2018; Karvonen et al. 2019; Nikitin et al. 2016; Pinna et al. 2017; Pozdniakova 2018; Schraven et al. 2021; Sinaeepourfard et al. 2016; Smigiel 2019; Valdez et al. 2018; Wu et al. 2018). This formed a number of contextual variegations and a complex tapestry of socio-technical visions, which further made the incarnations of the ideal of smart cities hard to identify and delineate. However, all these urban dynamics and their key factors contributed to stimulating research in the area of sustainable smart cities as manifested in the sharp rise in the number of publications from approximately 90 to 413, as shown in Fig. 2. As highlight in the Red cluster in Fig. 5, numerous applications of smart city technologies and solutions materialized to improve and advance environmental sustainability in relation to energy, transport, buildings, water, waste, climate, and others. The technologies involved infrastructures, systems, methods, models, techniques, and networks as part of the digital ecosystem of smart cities. The solutions developed encompassed the use of advanced decision support systems for management, control, optimization, efficiency, management, prediction, and forecasting.

*On the rise of AI and AIoT technologies and solutions and related models and techniques in environmentally sustainable smart cities* The year 2019 witnessed a dramatic increase in the use and adoption of AI or AIoT technologies and solutions in environmental sustainability, climate change, and smart cities. This followed the massive use and wide adoption of the IoT and Big Data technologies and solutions of smart cites during the period 2016–2018 (Ahmed et al. 2017; Ameer et al. 2018; Cheng et al. 2018; Hashem et al. 2016; Kumar and Prakash 2016; Nikitin et al. 2016; Perera et al. 2017; Rathore et al. 2016, 2018). The dramatic increase is consistent with the extant literature that reflects the general consensus on the complexity of the environmental degradation and climate change phenomena and thereby the growing need for more innovative, advanced, and immediate solutions based on the collective expertise in AI, Big Data, and IoT (Bibri et al. 2023; Nishant et al. 2020; Yigitcanlar et al. 2020). AI empowers the analysis of big data generated via the billions of the connected IoT devices in smart cities for generating useful knowledge in the form of applied intelligence. (González et al. 2018) shed light on the link between IoT and AI and its subsets of Machine Leaning (ML), Computer Vision (CV), Fuzzy Logic (FL), and Natural Language Processing (NLP). The combination of data-driven IoT and AI brings new improvements to the efficiency of IoT operations and data management and analytics models, which in turn enhance decision making and how humans and machines can interact toward structuring urban computing and intelligence systems for environmentally sustainable smart cities.

The computational capabilities of AI largely expand opportunities to understand, prevent, and handle environmental problems as well as transform decision-making processes. Therefore, AI models and techniques have gained traction and become widely applicable during the second period as part of AI and data-driven IoT solutions for environmental sustainability, climate change, and smart cities. For example, only during the period 2016–2019, there were more than 200 studies that applied AI to energy conservation and renewable energy (Nishant et al. 2020). Empirical AI research on environmental sustainability included water resources conservation based on ML using ANN, SVM, FL, Adaptive Neuro-Fuzzy Inference System (ANFIS), LR, and Key-Nearest Neighbors (KNN); energy conservation and renewable energy based on ML using ANN, FL, SVM, DT, Evolution Strategies (ES), Evolutionary Computing (EC), Batch-Normalization (BN), ANFIS, and pattern recognition; sustainable transportation based on ML using SVM, ANN, Decision Trees (DT), LR, and time series models; biodiversity conservation and ecosystem services based on ML using FL, GA, Artificial Intelligence for Ecosystems (ARIES), and BN (see Bibri et al. 2023 for a synthesis of relevant studies). For more examples of theoretical, empirical, and literature AI research on environmental sustainability and climate change during the second period, the interested reader might be directed to (Nishant et al. 2020). Concerning the same period, Bibri et al. (2023) synthesize several studies on climate change, which tend to focus largely on climate scenario analysis, climate adaptation and mitigation, and disaster management and resilience using several models (e.g., ML, Evolutionary Computing, FL) and techniques (e.g., ANN, BN, SVM, GA, neuro-fuzzy). Additionally, the authors synthesize a set of multiple theoretical and empirical studies on the application of AI and AIoT in smart cities based on ML, DL, CV, NLP, cognitive AI, and knowledge-based AI.

The growing interest in the innovative technologies and applied solutions of smart cities, coupled with their potential to tackle the environmental challenges of sustainability, resulted in an increase in research on a range of new themes related to environmentally sustainable smart cities, as illustrated in Fig. 5. The amalgamation of smart cities and sustainable cities in terms of their technologies, solutions, strategies, goals, and polices manifested itself into different approaches to urban development and models of urbanism focused on the environmental dimension of sustainability. These include, for example, data-driven smart cities (Sutherland and Cook 2017), smart cities for environmentally sustainable urban development (e.g., Angelidou et al. 2018), environmentally smarter cities (e.g., Bibri 2018b, 2019), and sustainable smart cities (e.g., Perera et al. 2017; Shruti et al. 2019; Silva et al. 2018).

##### Period 3 (2020–2021):

*Evolution of environmentally sustainable smart cities*. Figure 6 depicts the findings for the third period (2020–2021), which coincided with the outbreak of COVID-19. This crisis had some bearing on research activities in the fields of smart cities, environmental sustainability, and climate change. As shown in Fig. 2, there was a decrease in the quantity of the published works during the third period compared to the different years of the second period. Nevertheless, academic interest in the connection between smart cities, sustainable cities, and environmental sustainability, and data-driven technologies continued to steadily rise during the third period. This is illustrated in Fig. 2 through the upsurge of publications from 574 to 713. This is also reflected in Fig. 6 in the Green cluster, the Yellow cluster, the Blue cluster, and the Red cluster and related thematic focus. Important to note is that Fig. 6 represents only a 2-year period with 139 publications compared to Fig. 5 which represents a 4-year period with 323 publications. Moreover, there are some differences relating to the shift in the focus of the Yellow cluster, the Green cluster, and the Blue cluster across Figs. 6 compared to Fig. 5. This is attributed to the unexpected changes in the research activities of scholars and scientists and the funding priorities of grant agencies—triggered by COVID-19. With respect to the former, on the covidization of scientific work, (Cowen 2022)found that, during the period 2020–2021, COVID-19–related publications received 20% of all citations, and 98 of the 100 most-cited publication were associated with COVID-19. Further, as illustrated in Fig. 6, the Green cluster expands to include a large part of the Red cluster, compared to the second period. This indicates a shift in academic interest from focusing on the challenges related to climate change, environmental sustainability, environmental impacts, and air pollution toward focusing on the massive deployment and implementation of the technologies and solutions of smart cities—thanks to COVID-19. One implication of this is the wide adoption and integration of AI (the Red cluster), IoT, and big data analytics (the Yellow cluster) underlying the technological infrastructure of smart cities in relation to their amalgamation with sustainable cities (the Blue cluster).

In addition, the event of COVID-19 resulted in a further increase in the findings on existing and new topics. These are outlined in the analysis performed on the different publications as categorized into the four clusters. The Green cluster comprises publications focusing on climate change, emissions, air pollution, air quality, COVID-19, economic growth, circular economy, policy, water resources, green infrastructure, urbanization, resilience, and others. The Red cluster consists of publications targeting the implementation of the data-driven technologies and solutions of smart cities that could have positive impacts on both environmental sustainability and COVID-19 response through optimization, efficiency, management, performance, behavioral change, and others. The Yellow cluster represents publications covering the underlying infrastructure of smart cities, with new appearing topics, such as electric vehicles and waste management. Lastly, the Blue cluster is dedicated to publications focusing on broad issues related to sustainable cities, smart cities, and smart sustainable cities and their dimensions and synergies, with new appearing topics, such as digitalization and environmental indicators. Worth pointing out is that smart cities and sustainable cities graphically moved closer to one another compared to the second period, signalizing the materializing patterns of environmentally sustainable smart cities. More to this, smart cities became an integral part of the Blue cluster, which covers the broad issues related to smart sustainable cities during the second period. In a similar vein, sustainable cities became an integral part of the Red cluster, which was dedicated to the technologies and solutions of smart cities that could have implications for environmental sustainability during the second period.

Being associated with the rushed rollout and massive deployment of advanced technologies across major cities worldwide—and hence its ramifications on urban lives, urban dynamics, and urban futures, COVID-19 became a topic of high priority and impotence to address during the period 2020–2021. Against the backdrop of this study, this crisis attracted many scholars and practitioners in the fields of smart cities, environmental sustainability, and climate change, turning their attention to investigating or critically engaging with the topic from a variety of perspectives. In this regard, COVID-19 served as an opportunity to test the ability of smart city technologies and solutions to solve major societal issues, but also provides additional momentum to further develop and implement smart cities (Kunzmann 2020; Sharifi et al. 2021). Especially, its role in accelerating the transition toward smart cities and what this entails in terms of the large-scale process of digitalization was perceived to have implications for environmental sustainability. The digitalization trigged by the crisis significantly contributes to climate actions (Balogun et al. 2020; Dwivedi et al. 2022; Li et al. 2022)and can potentially reduce global emissions by approximately 15% by 2030 and additional 35% by shaping transformative systems and impacting economic decisions (Falk and Gaffany 2019). However, while extensive research has successfully reported on CO2 emissions reductions thanks to COVID-19 (Forster et al. 2020; Petetin et al. 2020; Rybarczyk and Zalakeviciute 2021), it remains substantially challenging to determine these reductions due to scale variations in atmospheric CO2 (Dacre et al. 2021). To put it differently, despite the estimated large decrease of the “global anthropogenic carbon dioxide (CO2) emissions…by up to 12% at the start of 2020 compared to recent years due to the COVID-19 related downturn in economic activity…, no reduction in the trend in background atmospheric CO2 concentrations has been detected” (Dacre et al. 2021).

*On the increasing adoption of AI or AIoT technologies and solutions and their expansion from environmental sustainability to sustainable smart cities. *The third period witnessed a marked intensification of the adoption of AI or AIoT technologies and solutions in smart cities and thus the emergence of several integrated models under the umbrella term of environmentally sustainable smart cities. As shown in Fig. 6, the wide application of AI or AIoT in the different areas of environmental sustainability and climate change reflects the continuous increase in academic interest in this emerging paradigm of urbanism. It was further widened due to the direct impacts of the spread of COVID-19 and the revitalization of AI (owing to new advances in Big Data and IoT) on the acceleration of the digital transformation of smart cities and its perceived role in optimizing resource efficiency and reducing emissions. Among the new topics that appeared in the Blue cluster during the third period is digitalization, which together with digitization constitutes the process of digital transformation that was abruptly accelerated as a result of the “new normal” prompted by COVID-19 and the readiness of rolling out AI or AIoT systems. With reference to data-driven smart cities, (Bibri et al. 2022) conceive of digitalization as “the ways in which urban processes are organized through and around digital technologies,” as well as enhanced by leveraging these “technologies and digitized data with respect to productivity, efficiency, and effectiveness through taking a process from a human-driven series of events to software-driven series of events. In short, it is the use of digital technologies to advance urban processes and provide value-generating and maximizing opportunities. Changes associated with digitalization are applied to data-driven smart cities as a social organization.” Smart cities have become immersed in a digital transformation that is enabled by the convergence of AI, IoT, and Big Data and its resulting processes of hyper-connectivity, datafication, and algorithmization (Calvillo et al. 2016). These processes have also affected sustainable cities in terms of accelerating their digital transformation, which has been steadily progressing since the mid-2010s. Further, as implied from the umbrella term of smart sustainable cities depicted in the Blue cluster in Fig. 6, the amalgamation of smart cities and sustainable cities was intensified during the period 2020–2021 with respect to their technologies, solutions, strategies, visions, and policies, with the first two dimensions being largely associated with the convergence of AI, IoT, Big Data in energy systems, power grid systems, transport systems, and environmental control systems.

Several AI models and techniques were used as part of AI and AIoT applications for environmental sustainability in the realm of smart cities, including DL, ANN, NN, SVM, and random forest. The slight difference graphically shown in Fig. 6 regarding the applied AI models and techniques compared to Fig. 5 could be attributed to the third period involving only two years compared to the second period which includes 4 years. Further, however, AI and AIoT applications involve, as shown in Fig. 6, management, optimization, efficiency, performance, design, demand response, consumption, modeling, simulation, and prediction.

Compared to Fig. 5, the empirical, theoretical, and literature research on AI and AIoT applications continued to rapidly evolve in the fields of environmental sustainability, climate change, and smart cities, with new emerging areas and themes. The research areas include energy conservation and renewable energy, sustainable transportation, water resources conservation, waste management, biodiversity and ecosystem services, pollution control, climate mitigation and scenario analysis, and disaster resilience and management. (Bibri et al. 2023) synthesize multiple studies reporting on the application of AI and AIoT in environmental sustainability, climate change, and smart cities during the period 2020–2022. Moreover, as shown in Fig. 6, the role of Blockchain technology lies in addressing challenges, managing resources, and improving services in smart cities in the context of environmental sustainability. It is not until 2019 onwards that it emerged as a new theme among the technologies and solutions of smart cities. Also, several applications of Blockchain technology involve its combination with the AI, IoT, and Big Data (Kouhizadeh and Sarkis 2018; Leal Filho et al. 2022; Parmentola et al. 2022; Wang and Qu 2019). Among the recent Blockchain technology applications are: green energy production and promotion (Parmentola et al. 2022); carbon emissions reductions (Liaqat et al. 2021); compliance with environmental standards (Mora et al. 2021)carbon credits and reward schemes (Mora et al. 2021); water pollution reduction and water resources conservation (Parmentola et al. 2022); and water and wastewater management and the nexus between various natural resources in cities (Blasi et al. 2022; Mora et al. 2021; Parmentola et al. 2022; Vinuesa et al. 2020).

As to the amalgamation of smart cities and sustainable cities during the third period, the environmental dimension of sustainability became one of the key driving factors for the innovative technologies and applied solutions of smart cities. This is also illustrated in Fig. 6, where the scholarly focus shifted from criticizing the fragmentation of smart cities and sustainable cities toward developing more integrated models of urbanism. Among the prominent models that emerged during and beyond the period 2020–2021 include: sustainable and smart cities (Makani et al. 2022), eco-friendly and sustainable smart cities (Almalki et al. 2021), sustainable smart cities (Al-Dabbagh 2022; Kim et al. 2021; Saravanan and Sakthinathan 2021; Tripathi et al. 2022), environmentally sustainable smart cities (Singh et al. 2022), environmentally data-driven sustainable smart cities (Bibri and Krogstie 2020), circular smart cities (e.g., (Abou Baker et al. 2021; Núñez-Cacho et al. 2022). circular smart and connected cities (Sertyesilisik 2023), circular virtual smart cities (Allam et al. 2022b; Musti 2020) and artificially intelligent and sustainable smart cities (Gourisaria et al. 2023).

## Discussion

### The changing elements and interlinked developments reshaping the landscape of smart cities

The prospect that AI, IoT, and Big Data technologies will instigate massive changes in smart cities is fast becoming the new reality due to the acceleration of their digitalization and digitization. The digital transformation being generated by the convergence of these innovative technologies is seen to hold great potential to tackle the problematicity surrounding the planning and development of smart cities in response to the decabornization and climate change emergencies. In parallel, the need to streamline the transition to environmental sustainability and mitigate the impacts of climate change has given rise to the phenomenon of environmentally sustainable smart cities. As natural resources have become highly on demand due to the escalating rate of urbanization and urban growth, smart cities are making huge efforts to find new and innovative ways to reduce their consumption of resources and thus lessen their impacts on the environment. This is reflected in the growth of knowledge of the relevant facets of sustainable smart cities from the mid-2010s onwards. This growth is manifested in the marked upsurge in the scholarly output on this multifaceted topic, with new terms being coined and new themes and areas being explored in response to the increasing need for more integrated approaches to sustainable urban development. The growing number of the academic works devoted to the topic of environmentally sustainable smart cities has coincided with the rise of the databases tracking this paradigm of urbanism in terms of its scientific, technological, environmental, and institutional dimensions.

Academic interest in the topic of smart sustainable cities started to increase around 2014 to 2016, grew exponentially in 2017, and evolved rapidly afterwards. One of the main factors behind the knowledge growth in this area is the rapid advancement of AI, IoT, and Big Data technologies and their novel applications, in addition to the general consensus on the urgency to tackle the challenges of environmental sustainability and climate change. The first period witnessed the materialization of sustainable cities in the early 1990s in the wake of the widespread diffusion of sustainable development, the materialization of smart cities in the early 2000s following the acceptance of smart growth movement, and the materialization of smart sustainable cities around the mid-2010s in response to the SDGs agenda and the rising concerns over urbanization. The second period marked the materialization of sustainable smart cities and a few related integrated models of urbanism in light of the advancement of the IoT and Big Data technologies and solutions. The third period heralded the emergence of environmentally sustainable smart cities and several related integrated models of urbanism under different labels—due mainly to the convergence of AI, IoT, and Big Data technologies and its consequential datafied, algorithmized, and platformized penetrative patterns. The essence of this new paradigm of urbanism lies in leveraging the collective power and multifaceted potential of these advanced technologies in making progress toward achieving the goals of environmental sustainability. While research on the relationship between smart cities, sustainable cities, environmental sustainability, and data-driven technologies is still in its infancy, the convergence of these interlinked developments is rapidly gaining traction as a strategic approach to sustainable urban development.

Beyond the shadow of a doubt, sustainable development and technological advancement have, at varying degrees, inspired different urban scholars, scientists, and practitioners in the pursuance of explorations and investigations to unlock numerous opportunities to improve the health of the city and the quality of life of its citizens. This has more often than not taken the form of integrating sustainability strategies and technology solutions in the field of urban planning and development in the attempt to slash energy consumption, reduce pollution, lower material use, and minimize waste. In particular, the notion of sustainable development has, since the early 1990s, motivated scholars and practitioners to device more integrated and holistic frameworks for urban development that can rise up to the challenges of environmental sustainability head on through enhancing the performance of urban systems, connecting urban infrastructures, coordinating urban domains, and revitalizing underdeveloped areas. In recent years, ICT-based innovations have undeniably become pivotal in facilitating the practical endeavors of urban sustainability and its transformative changes. This has led to the emergence of an alternative paradigm to sustainable cities and smart cities—sustainable smart cities. This paradigm further interplaying with the recent global factors, notably the appearance of COVID-19, the digital transformation of urban societies, and the accelerated transition to smart cities, has brought about the phenomenon of environmentally sustainable smart cities. While this phenomenon has many faces, this study is concerned with the integration of the emerging data-driven technologies and solutions of smart cities in the field of environmental sustainability with the green technologies and strategies of sustainable cities. As such, it expands on the previous works done on environmentally smart sustainable cities that emphasize the innovative and synergistic potential of IoT and Big Data in improving and advancing environmental sustainability (Bibri and Krogstie 2020) by adding AI to enhance this innovative and synergistic potential—under AIoT—for achieving optimal outcomes in the different areas of environmental sustainability. This is motivated by the recent advances in AI and its potential to provide new solutions for tackling environmental challenges.

### The impact of AI, IoT, and big data technologies on the dynamics of smart cities

AI, IoT, and Big Data technologies have been instrumental in pushing smart cities to rethink their strategies for sustainable development. This is reflected in the increased interest among scholars to develop new approaches and solutions that could make smart cities environmentally more sustainable. In recent years, the rise of Big Data and computing power has empowered AI, and its new generation is rapidly expanding and becoming “an attractive topic for research” (Duan et al. 2019). As consistent with the findings for the second and third periods, ANN, Deep Neural Network (DNN), SVM, GA, LR, and DT are among the prominent AI techniques that gained strong traction in recent years (Şerban and Lytras 2020; Shrestha and Mahmood 2019), so are ML, DL, and cognitive AI models in relation to the planning and development of smart cities (Kamrowska-Załuska 2021; Şerban and Lytras 2020). Similarly, AI has empowered the analysis of the vast troves of data generated via the IoT infrastructure in smart cities. Consequently, a great deal of research and innovation was witnessed in the field of AI in relation to environmental sustainability, climate change, and smart cities (e.g., (Nishant et al. 2020); (Leal Filho et al. 2022; Vinuesa et al. 2020; Yigitcanlar and Cugurullo 2020; Yigitcanlar et al. 2020) during the second and third periods. These new developments in AI triggered major breakthroughs with clear consequences across all domains of smart cities. As a result, despite the COVID-19 crisis and its impact on the research activities relating to smart cities, the quantity of the published works continued to rise during the third period. In parallel, AI or AIoT systems were massively deployed across smart cities to trial new modus operandi for learning, adaptation, and network effect purposes, as well as to test and institute new public health and environmental reforms through knowledge and policy networks. In relation to public health, (Adly et al. 2020) discuss the application of AI for preventing and controlling COVID-19 and intelligent IoT for COVID-19 data gathering and integration. In the face of COVID-19, AI researchers have applied ML/DL to diagnose the disease as part of imaging intelligence-based medicine (Desai et al. 2020)Concerning the environment, the digital transformation of urban societies intensified by COVID-19 and accelerated by the adoption of AI or AIoT solutions for environmental sustainability and climate change during the third period were largely perceived to dramatically change how natural resources could be regulated and managed to prevent environmental problems in the context of smart cities. This in turn induced scholars to propose new frameworks for integrating smart cities, sustainable cities, environmental sustainability, and advanced ICT, thereby giving rise to the phenomenon of environmentally sustainable smart cities.

Being at the core of advanced analytical systems, AI is also reshaping how the IoT infrastructure operates in terms of data collection and aggregation, big data analytics functions in terms of data management and processing, and decision-making is performed in terms of support, automation, and autonomization. The latter entails replacing human intellect instead of expanding it in future urbanism. AI systems can serve either to assist or replace the human decision makers (Duan et al. 2019). However, IoT can make use and leverage the power of AI via the connectivity of devices and the intelligence of data by rendering their collection more dynamic for efficient analysis. Current trends show that AIoT will become the predominant paradigm of urban computing and intelligence in the near future. This stems from the new capabilities enabled by AI systems as having access to large volumes of the data collected via IoT and organized in large datasets to perform new complex analyses that were inconceivable not long time ago due to the inability to harvest and aggregate data in a real-time fashion, especially in relation to urban planning and development. Accordingly, the availability of huge amounts of data in smart cities thanks to IoT offers prospects of advancing environmental sustainability through enhanced decision-making processes thanks to AI pertaining to the optimal use of natural resources. However, the development and implementation of environmentally sustainable smart cities will require continuous innovations in the so-called cutting-edge technologies based on the projected trends for the next generation of AI, IoT, and Big Data.

### Environmental costs of AI, IoT, and big data technologies

AI, IoT, and Big Data technologies and solutions are associated with huge environmental costs in smart cities. This is a daunting challenge to deal with and a special conundrum that should not be overlooked. According to a recent review study conducted by (Bibri et al. 2023), while these advanced technologies provide new and largely expand opportunities to better understand, handle, and prevent environmental problems, they are challenged by their environmental risks that need to be addressed and overcome. The use of AI applications to enhance urban efficiencies involves struggles in accomplishing the transformative changes to smart cities due to “the short-sighted, technologically determined, and reductionist AI approaches being applied to complex…problems” (Yigitcanlar et al. 2021). While “we are repeatedly told that AI will help us to solve some of the world's biggest challenges,… Public discourse on AI systematically avoids considering AI’s environmental costs…If we want to stand a chance at tackling the Climate Emergency, then we have to stop avoiding addressing the environmental problems generated by AI” (Brevini 2020). AI involves establishing heavy energy dependency and increasing carbon footprints (Dauvergne 2021) as a result of the externalities of its development, use, application, and disposal (e.g., (Almalki et al. 2021; Kaplan and Haenlein 2019; Leal Filho et al. 2022). The systems, machines, and infrastructures associated with AI “deplete scarce resources in their production, consumption, and disposal, thus increasing the amounts of energy in their use, and exacerbate problems of waste and pollution” (Brevini 2020). It is estimated that the global electricity demand of advanced technologies (AI, IoT, and Big Data) could go up to 20% compared to around 1% today (Vinuesa et al. 2020). In particular, large data centers, which provide massive computational resources required to compute, analyze, index, and mine AI-derived knowledge, devour energy. This pattern of consumption compromises the affordable energy and climate action targets of sustainable development. Indeed, the environmental costs of AI models and big data analytics “are not accounted for when developing new policies on AI” (Brevini 2020)In fact, there is ambiguity on how AI may specifically contribute to environmental sustainability in the realm of smart cities. In more detail, there is a risk of a mismatch between the environmental targets of smart cities and the opportunities offered by AI, IoT, and Big Data technologies, and therefore, their green growth and eco-friendly design are of critical importance to mitigate such risk (Bibri et al. 2023). Regardless, there is a whole established academic and policy discourse that tends to mythologize and evangelize AI as a panacea for environmental and climate change challenges, and even as an inevitable reality in modern society. In this regard, promises are often made without really providing a strategic roadmap to environmental change, thereby the rhetorical inclination of dominant discourses around AI. AI has been enshrined in dominant narratives as “the battleground for global dominance and progress…hailed as the key marker of victory…and the opportunity of the future—and thereby selling it as a virtue and a public good” (Brevini 2020).

### Ethical risks and regulatory conundrums of AI technology

The ethical risks of AI are one of the key topics in academic and policy debates, and constitute the most difficult conundrum to solve. They include privacy, security, trust, bias, unfairness, lack of transparency, and social inequality (Ahmad et al. 2020; Chen et al. 2020; Hoffmann 2019; Larsson and Heintz 2020; Yigitcanlar et al. 2020). Most of these issues are at the core of Explainable AI (XAI), which is one of the most heavily debated topics when it comes to the application of AI in smart cities. Ghonge et al. (2023) address a number of use cases of XAI as well as its impacts and open challenges in smart city applications. Moreover, privacy, cybersecurity, and trust issues have been extensively debated in relation to IoT, and most of the technological safeguards developed to mitigate them have failed (Bibri 2015)and are likely to continue to fail due to inherent flaws. Especially, privacy is set to be exacerbated due to the inadequacy of data governance practices, where priority is more often than not given to efficiencies over regulatory requirements. Data privacy has been long and extensively debated in legislatures, resulting in “data protection oversight agencies and a modest level of jurisprudence,” while provisions facilitating all forms of digital surveillance are voluminous (Clarke and Greenleaf 2017). In view of that, coupled with the AI-based technologies being still in their incipient stages, it is necessary to devise more effective regulatory and governance measures to ensure they will not undermine ethical and humanistic values. Ensuring that AI is human centered requires government interventions (Mhlanga 2022), and ethical standard and regular auditing are key to maintain compliance with regulatory frameworks governing the use and adoption of AI (Truby 2020). It has become of critical importance to develop “responsible and ethical AI” before it is too late (Barredo Arrieta et al. 2020); (Burton et al. 2020); (Matthias 2004)This approach also relates to XAI challenges (Barredo Arrieta et al. 2020). There is a need for well-regulated and responsible AI systems that should protect public values.

Regulatory policies and poly-centric governance systems are more needed than ever under the current circumstances of democratic decay and privacy loss. This is to ensure that AI systems take into account algorithmic fairness, transparency, accountability, and safety as core values and principles of their designs, thereby upholding procedural and algorithmic justices—instead of outsourcing these ethical and social values to the global technology sector. Incidentally, COVID-19 has worsened the effects of big tech companies in terms of their interference in data policy and data governance as a result of the accelerated adoption of digital technologies (Li et al. 2022), adding to its rising economic, environmental, and social impacts as compounded by the Russia-Ukraine war (Allam et al. 2022a). Engaging and consulting multiple stakeholders could ensure that AI solutions balance between the environmental and social goals of sustainability while mitigating the risks of the economic and political biases and ideologies inherent in data-driven decision-making processes. One of the solutions that could offer a fair ground for engaging multiple stakeholders in smart cities and thus enhancing the prospect of their contribution to the SDGs is designing standard and interoperable formats for secure data collection and exchange (Liaqat et al. 2021)—supported with privacy-enhancing mechanisms.

## Recommendations for future research

There are unique opportunities for advancing the planning and development of emerging environmentally sustainable smart cities to achieve better performance outcomes and thus the targets of the SDG 11. As this is an emerging model of sustainable urban development that is attempting to unlock and exploit the multifaceted potential of the converging IoT, Big Data, and AI technologies and solutions, there are many questions that need to be addressed to secure the expected benefits of environmental sustainability. This relates to a number of gaps in our knowledge on emerging environmentally sustainable smart cities that follow from our results and their discussion. Below are some of the recommendations we deem relevant for further research:

- It would be helpful to conduct an in-depth exploration of the other AI models than those mostly applied in relation to IoT and Big Data in the field of environmental sustainability and their strengths and weaknesses. The deficiencies inherent in the existing ML/DL models with respect to decision-making processes in large-scale urban AI system warrant further research. Research might also explore why some areas of environmental sustainability are given more attention than others in AI research. It would be useful to investigate other driving factors behind the emergence of environmentally sustainable smart cities than those identified in this study.
- The trade-offs of the adoption of AI, IoT, and Big Data technologies and solutions in environmentally sustainable smart cities would be of high importance to examine for generating complementary and conflicting perspectives and insights. This would be best carried out through systematic reviews to deliver a clear and comprehensive overview of relevant, trustworthy empirical evidence and identify further gaps in the current understanding of this emerging paradigm of urbanism.
- It would be interesting to analyze in more depth how environmentally sustainable smart cities could balance between the environmental, economic, and social objectives of sustainable development by leveraging the convergence of AI, IoT, and Big Data technologies and their collective expertise.
- Given that the convergence of AI, IoT, and Big Data technologies is still in its incipient stage with respect to generating transformative changes in environmentally sustainable smart cities, there is a need to critically engage with:
  - How regulatory frameworks and governance systems are responding to critical debates over the need for the responsible development and adoption of AI or AIoT systems.
  - How the goals of environmentally sustainable smart cities and the opportunities offered by their converging IoT, Big Data, and AI technologies and solutions could be aligned.
  - What bottlenecks and barriers exist in the development of holistic models for containing the environmental risks of AI, IoT, and Big Data technologies.
- More methodological work is needed on how to robustly capture the positive impacts and outcomes of emerging environmentally sustainable smart cities, including further enviro-economic and social critical analyses. These are of increasing importance to the field of green and public policy.
- It would be beneficial to carry out a full cost–benefit analysis of AI, IoT, and Big Data technologies. Despite being methodologically challenging, it would be highly useful to conduct some in-depth studies to quantify the impact of these advanced technologies on not only key environmental but also economic and social indicators in the realm of sustainable smart cities as a holistic approach to sustainable development.

## Conclusion

The world has recently experienced unprecedented challenges and increased uncertainties, making it more challenging for smart cities to improve their environmental efficiency and to contribute to actual progress toward reaching the environmental targets of the SDG 11. This has, together with other global and technological factors, led to the materialization of environmentally sustainable smart cities as an umbrella term for several models of urbanism focusing mainly on the environmental dimension of sustainability. This new paradigm of sustainable urban development entails integrating the data-driven technologies and solutions of smart cities and the green technologies and strategies of sustainable cities. The idea revolves around leveraging the collective expertise in AI, IoT, and Big Data in tackling the challenges of environmental sustainability and climate change. The technologies and solutions of smart cities are creating many new opportunities for better understanding, preventing, and handling environmental problems as well as enhancing decision-making processes.

This study explored the key research trends and driving factors of environmentally sustainable smart cities and maps their thematic evolution. In addition, it examined the fragmentation, amalgamation, and transition of their underlying models of urbanism as well as their converging AI, IoT, and Big Data technologies and solutions. The results showed that environmentally sustainable smart cities are a rapidly growing trend that markedly escalated during the second and third periods—due to the acceleration of the digitalization and decarbonization agendas—thanks to COVID-19 and the rapid advancement of data-driven technologies. The analysis also revealed that while the overall priority research topics have been dynamic over time—some AI models and techniques and environmental sustainability areas have received more attention than others. The evidence synthesized indicated that the increasing criticism of the fragmentation of smart cities and sustainable cities, the diffusion of the SDGs agenda, and the recent dominance of advanced ICT have significantly impacted the materialization of environmentally sustainable smart cities, thereby influencing the landscape and dynamics of smart cities. It also suggested that the convergence of AI, IoT, and Big Data technologies provides new approaches to tackling the challenges of environmental sustainability. This convergence is of crucial importance to make critical urban infrastructure resource-efficient, low-emission, and resilient by making progress toward reaching the environmental targets of the SDG 11 related to energy, transport, waste, water, biodiversity, and climate. However, the converging data-driven technologies involve environmental costs and pose ethical risks and special regulatory conundrums.

While the analysis and synthesis has allowed us to achieve the overall aim of the study, there are some limitations that need to be highlighted so to be addressed in future research. One key limitation lies in the current study being based solely on peer-reviewed literature. It would be useful to consider grey literature to gain and provide more comprehensive and clear insights. Thus, future research may combine both categories of literature or should prioritize grey literature for better knowledge outcomes, as well as include other databases to have a sufficiently reasonable and representative form of results. Especially, the dynamics of smart cities in terms of their converging AI, IoT, and Big Data technologies and their recent advancements as shaping forces essentially occur as coordinated actions of local governments and international organizations. Therefore, research produced by these entities, including reports, working and evaluation papers, and planning and policy documents, would add value to knowledge in future research. In addition, given the marked effects of COVID-19 on the intensified digitalization of smart cities and thus the massive roll out of advanced technologies, it would be useful and relevant to conduct research to establish the post-pandemic landscape of publications on emerging environmentally sustainable smart cities. This would map out or trace how both COVID-19 and AI innovations have impacted on urban policy and governance in relation to environmental sustainability. A comparative study of these two influential factors would also be beneficial. Further research on the large-scale digital transformation of urban societies might serve to highlight the role of recent scientific and technological advances in the infusion of data-driven solutions as the new frontiers in urban environmental policy and governance systems.

We anticipate this study will stimulate debates over the substantive effects of the converging AI, IoT, Big Data technologies on the future form of sustainable urban development, enhance the understanding of the multifaceted nature of the flourishing field of environmentally sustainable smart cities, and provides grounding for spurring groundbreaking research and large-scale implementations. Especially, this area is in infancy research and the early stages of its development, with great potential to bring about meaningful urban transformations based on the responsible development and implementation of the AI, IoT, and Big Data technologies and solutions of smart cities.

## Data Availability

Not applicable.

## Appendix

### The search string

TS = ((“smart” OR “intelligent” OR “digital” OR “digitali*ation” OR “Information and communication technolog*” OR “ict” OR “information technology” OR “internet of things” OR “iot” OR “artificial intelligence” OR “AI” OR “machine learning” OR “deep learning” OR “blockchain” OR “virtual reality” OR “VR” OR “augmented reality” OR “AR” OR “cloud computing” OR “big data” OR “5G” OR “6G” OR “industry 4*” OR “society 5*” OR “robotic*” OR “automation” OR “automated” OR “city brain” OR “metaverse” OR “autonomous vehicle*” OR “autonomous machine*” OR “neural machine translation” OR “long short term memory” OR “bidirectional encoder representation from transformers” OR “linear discriminant analysis” OR “neural network” OR “natural language processing”) NEAR/11 ("city" OR "cities" OR "urban") NEAR/11 (“environmental*” OR “ecology” OR “ecological” OR “natural environment*” OR “natural conservation” OR “natural resource*” OR “natural preservation” OR “nature protection” OR “ecosystem*” OR “biodiversity” OR “species habitat” OR “species protection” OR “climat* change*” OR “waste” OR “wastewater” OR “energy” OR “energies” OR “green*” OR “forest” OR “tree cover” OR “blue infrastructure” OR “urban agriculture” OR “urban farming” OR “transport*” OR “traffic” OR “mobilit*” OR “building*” OR “parking” OR “pollution” OR “emission*” OR “carbon” OR “co2” OR “NOx” OR “SO2” OR “N2O” OR “PM2.5” OR “PM10” OR “ozone” OR “air quality” OR “water” OR “wetland*” OR "sustainable environment*" OR “smart sustainab*” OR “sustainab* smart” OR “urban sustainability” OR “sustainable urban”)).

## Abbreviations

AI: Artificial intelligence
AIoT: Artificial Intelligence of Things
ANFIS: Adaptive neuro-fuzzy inference system
ANN: Artificial neural network
ARIES: Artificial Intelligence for Ecosystems
BN: Batch-normalization
CO2: Carbon dioxide
COVID-19: Corona Virus Disease 2019
CV: Computer vision
DL: Deep learning
DNN: Deep neural networks
DT: Decision trees
EC: Evolutionary computing
FL: Fuzzy logic
GA: Genetic algorithm
ICT: Information and Communication Technology
IoT: Internet of Things
Maas: Mobility-as-a-service
ML: Machine learning
NLP: Natural language processing
SDGs: Sustainable development goals
SVM: Support vector machine
